# METAnnotatorX2: a Comprehensive Tool for Deep and Shallow Metagenomic Data Set Analyses

**DOI:** 10.1128/mSystems.00583-21

**Published:** 2021-06-29

**Authors:** Christian Milani, Gabriele Andrea Lugli, Federico Fontana, Leonardo Mancabelli, Giulia Alessandri, Giulia Longhi, Rosaria Anzalone, Alice Viappiani, Francesca Turroni, Douwe van Sinderen, Marco Ventura

**Affiliations:** a Laboratory of Probiogenomics, Department of Chemistry, Life Sciences, and Environmental Sustainability, University of Parma, Parma, Italy; b Microbiome Research Hub, University of Parma, Parma, Italy; c GenProbio srl, Parma, Italy; d APC Microbiome Ireland and School of Microbiology, Bioscience Institute, National University of Ireland, Cork, Ireland; University of Copenhagen

**Keywords:** metagenomics, shallow, deep, taxonomy, functional profiling

## Abstract

The use of bioinformatic tools for read-based taxonomic and functional analyses of metagenomic data sets, including their assembly and management, is rather fragmentary due to the absence of an accepted gold standard. Moreover, most currently available software tools need input of millions of reads and rely on approximations in data analysis in order to reduce computing times. These issues result in suboptimal results in terms of accuracy, sensitivity, and specificity when used either for the reconstruction of taxonomic or functional profiles through read analysis or analysis of genomes reconstructed by metagenomic assembly. Moreover, the recent introduction of novel DNA sequencing technologies that generate long reads, such as Nanopore and PacBio, represent a valuable data resource that still suffers from a lack of dedicated tools to perform integrated hybrid analysis alongside short read data. In order to overcome these limitations, here we describe a comprehensive bioinformatic platform, METAnnotatorX2, aimed at providing an optimized user-friendly resource which maximizes output quality, while also allowing user-specific adaptation of the pipeline and straightforward integrated analysis of both short and long read data. To further improve performance quality and accuracy of taxonomic assignment of reads and contigs, custom preprocessed and taxonomically revised genomic databases for viruses, prokaryotes, and various eukaryotes were developed. The performance of METAnnotatorX2 was tested by analysis of artificial data sets encompassing viral, archaeal, bacterial, and eukaryotic (fungal) sequence reads that simulate different biological matrices. Moreover, real biological samples were employed to validate *in silico* results.

**IMPORTANCE** We developed a novel tool, i.e., METAnnotatorX2, that includes a number of new advanced features for analysis of deep and shallow metagenomic data sets and is accompanied by (regularly updated) customized databases for archaea, bacteria, fungi, protists, and viruses. Both software and databases were developed so as to maximize sensitivity and specificity while including support for shallow metagenomic data sets. Through extensive tests performed on Illumina and Nanopore artificial data sets, we demonstrated the high performance of the software to not only extract taxonomic and functional information from sequence reads but also to assemble and process genomes from metagenomic data. The robustness of these functionalities was validated using “real-life” data sets obtained from Illumina and Nanopore sequencing of biological samples. Furthermore, the performance of METAnnotatorX2 was compared to other available software tools for analysis of shotgun metagenomics data.

## INTRODUCTION

Microbial communities exert key biological functions in a wide range of host-associated and environmental ecological niches spanning from soil and water to specific tissues and body sites in plants and animals (1–6). The ubiquitous presence of bacteria in natural and artificial environments explains why microbial populations are now extensively studied for their relevance pertaining to many aspects of human life, including not only health and disease conditions but also industrial, agricultural, and environmental settings (7).

Due to the increasing interest in microbial ecology, approaches based on next-generation sequencing (NGS) have been developed to obtain taxonomic, functional, and genomic information. These techniques can be divided into two main branches, being either amplicon based or shotgun sequencing based. The methodologies that rely on PCR-based amplicons rapidly became the most popular due to their lower cost, availability of comprehensive bioinformatic tools such as mothur and Qiime (8, 9) for data analysis, and the fact that they did not require extensive computing resources. Though the latter approach is widely used to reconstruct accurate taxonomic profiles, it is limited in that it generally makes taxonomic assignments as far as the genus level (10, 11). Recent methodological implementations (such as Dada2 to call amplicon sequence variants [ASVs] and uSearch to call zero-radius operational taxonomic units [zOTUs]) can lead to unique amplicon sequences that allow assignment to species level, but this is highly dependent on the amplified variable region of the 16S rRNA gene and on its taxon-specific variability in closely related species. While long read sequencing of the full-length 16S gene is still in its early stages of development, approaches based on shotgun metagenomics sequencing are now becoming increasingly popular. In fact, shotgun metagenomic data provide reliable information for reconstruction of taxonomic profiles that are accurate down to the species level (10–12). In addition, since shotgun metagenomic sequencing of complex bacterial populations covers the overall genetic content of constituent microorganisms, this approach can be used to generate functional information by screening for specific genes of interest and/or evaluate presence/absence of metabolic pathways. Moreover, assembly of metagenomic data sets allows the reconstruction of draft genomes of the most abundant species (12–14). In this context, introduction of long read sequencing, such as that generated by PacBio and Nanopore sequencing platforms, now offers the opportunity to drastically improve quality and completeness of metagenomic assemblies (13) and may allow the reconstruction of high-quality draft genomes from metagenomic data sets, being suitable for genomic and comparative genomic analyses.

While the vast majority of metagenomic data sets provided by next-generation sequencers has the potential to provide comprehensive biological information, it comes with the limitation of requiring extensive computing resources and associated specialist software. For this reason, currently available bioinformatic tools tend to rely on approximations for read-based taxonomic or functional classifications, such as identification of genus/species-specific marker genes or mapping of reads on reference genomes (15–18). The downside of such approaches is that accuracy of the results is directly correlated with the availability of reference genomes with sufficient sequence identity to those constituting the analyzed microbial populations (19). While this has only a minor impact on profiling of eukaryotes, due to limited sequence variability among strains, it may cause a drastic drop in taxonomic assignment accuracy and/or failure in detection when such approaches are used for profiling complex bacterial or viral communities. Furthermore, reference genomic databases suffer from taxonomic inconsistencies, as revealed by NCBI genome-based average nucleotide identity (ANI) analyses (20), thereby contributing to the loss of accuracy of taxonomic assignments.

To address these issues, we developed METAnnotatorX2, a tool for read- and assembly-based analysis of metagenomic data sets, including processing of hybrid data sets composed of a combination of short and long reads. The METAnnotatorX2 platform required a complete overhaul of its predecessor METAnnotatorX (12) so as to provide a user-friendly tool for taxonomic and functional profiling of metagenomic short read data sets through a novel approach that maximizes sensitivity and specificity by relying on improved databases and custom scripts. Moreover, METAnnotatorX2 represents a comprehensive easy-to-use pipeline for assembly of short, long, or hybrid read-based metagenomic data sets and species-specific genome reconstruction in GenBank format with gene prediction and associated functional annotation based on the MEGAnnotator pipeline (21).

## RESULTS AND DISCUSSION

### The METAnnotatorX2 pipeline.

**FIG 1:**
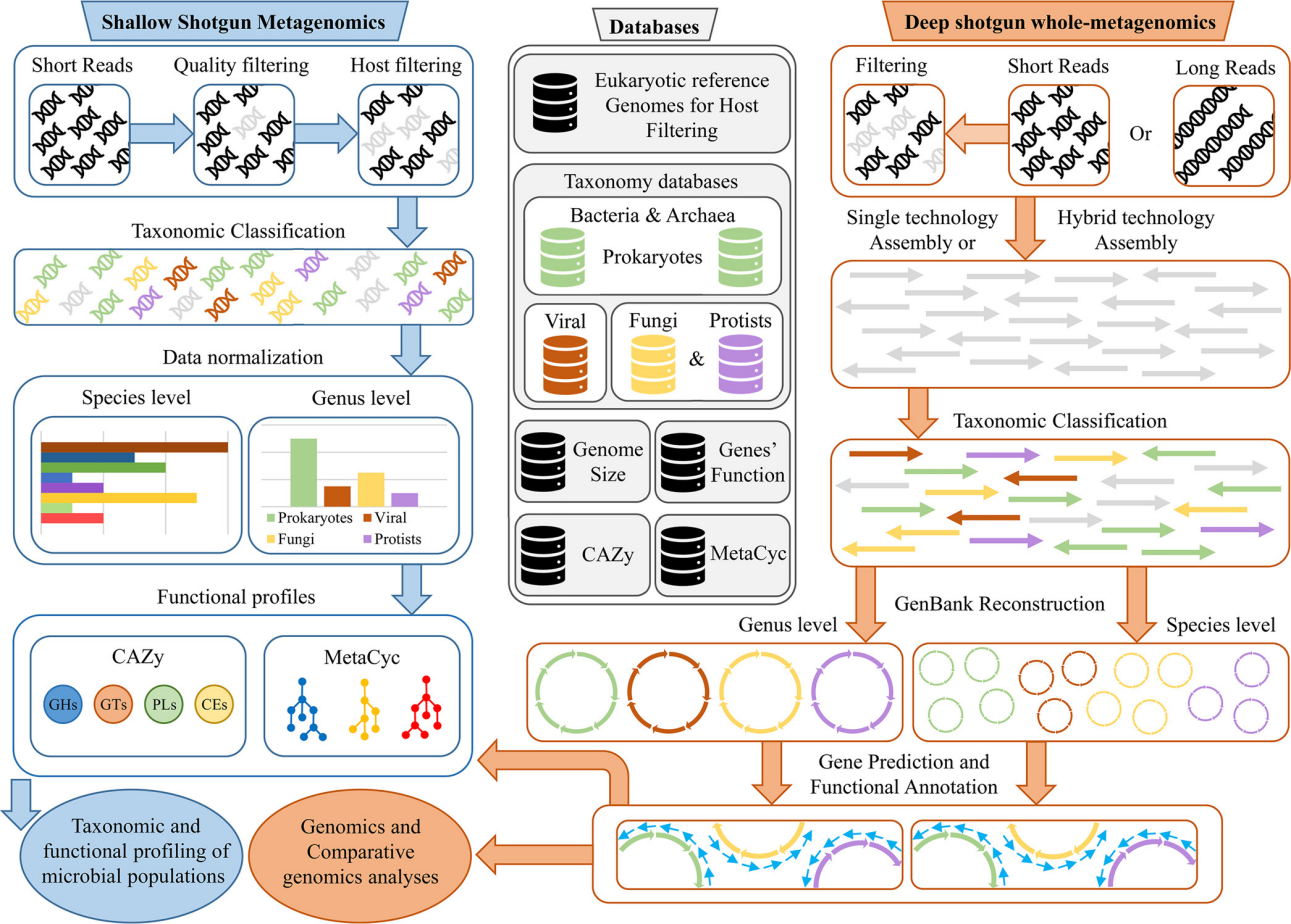
Schematic representation of read-based and assembly-based pipelines offered by METAnnotatorX2 for analysis of shotgun metagenomics data.

The current range of available bioinformatic tools for analysis of shotgun metagenomic data sets relies on approximations for taxonomic or functional classifications based on reads, such as identification of genus/species-specific marker genes or mapping of the reads on reference genomes (22, 23). The drawback of these approaches is that quality and reliability of the results are dependent on the availability of reference genomes with high sequence identity to those constituting the analyzed microbial populations, i.e., phylogenetically closely related strains with limited unique genomic regions (22, 23). Taxonomic classification based on homology searches through local alignments of reads in publicly available databases represents a superior solution in terms of sensitivity and overall accuracy, yet this approach has been avoided in the past due to longer computing times. However, recent advances in computer hardware, e.g., introduction of central processing units (CPUs) with an increased number of computing cores, and development of local alignment software, e.g., MegaBLAST (24), now makes this approach feasible for day-to-day applications. Moreover, shallow metagenomics, i.e., analysis of a limited number of reads (500,000 or lower), generated by shotgun metagenomic sequencing, has become popular as a cost-effective alternative to 16S rRNA gene-based microbial profiling as it allows species-level accuracy with similar requirements in terms of sequencing data (11). For this reason, we developed a novel application, METAnnotatorX2, based on the previously released METAnnotatorX tool (12). METAnnotatorX2 encompasses a complete pipeline for taxonomic and functional analyses of short reads and a pipeline for metagenomic assembly followed by taxonomic classification of contigs as well as gene prediction and functional annotation (Fig. 1). The technological improvements of METAnnotatorX2 compared to METAnnotatorX are outlined in Table S1 in the supplemental material.

10.1128/mSystems.00583-21.4 TABLE S1 Improvements offered by METAnnotatorX2 with respect to the previous version METAnnotatorX. Download Table S1, XLSX file, 0.01 MB. Copyright © 2021 Milani et al. 2021 Milani et al. https://creativecommons.org/licenses/by/4.0/ This content is distributed under the terms of the Creative Commons Attribution 4.0 International license.

In detail, input metagenomic data in .fastq format undergoes an initial quality filtering step. Subsequently, the user is given the possibility to perform removal of host DNA in case the analyzed data set refers to host-associated microbial populations. The latter filtering step is a novel implementation of the METAnnotatorX2 with respect to its predecessor, allowing the user to directly process raw sequencing data with METAnnotatorX2 instead of processing the data with additional external software aimed at data management. Retained sequences can then be used for read-based and assembly-based analyses. Regarding read-based analyses, METAnnotatorX2 can perform a MegaBLAST local alignment of reads to preprocessed databases designed to provide high accuracy taxonomic reconstruction. Although the accuracy of homology-based searches is higher than search applications based on marker genes and/or read mapping, the use of publicly available, unprocessed databases may negatively impact the obtained results, especially for prokaryote profiling, as revealed by average nucleotide identity analysis of the NCBI genome database (20). Thus, we developed specific databases for eukaryotes (fungi, encompassing yeasts, and protists), bacteria, archaea, and viruses that are available for download separately or together as a comprehensive database for microbial profiling (see below for details). Release of updated databases is planned at 6-month intervals. Reads with a nucleotide identity of >94% to reference genomes are classified at the species level, while reads with a lower percentage identity are classified at the genus level as undefined species. These cutoffs are those generally employed for the ANI taxonomic assignment of genomes (20, 25). Notably, reads showing equal best hits of nucleotide identity to multiple species are automatically submitted to least common ancestor (LCA) prediction using the JGI BBTools Taxonomy Server (https://taxonomy.jgi-psf.org/) in order to provide the most updated LCA assignment. This LCA step is disabled for reads that match against sequences in the viral database due to high sequence identity in publicly available viral genomes. The previous release of METAnnotatorX performed read-based taxonomic classification using the best hits provided by the RAPSearch2 tool against unprocessed gene-based RefSeq databases. Here, the METAnnotatorX2 read-based algorithm takes advantage of a hundred hits against filtered and taxonomically validated NCBI RefSeq genomes to generate an exhaustive taxonomic classification of each read. In addition to standard taxonomic profiles for viruses, prokaryotes, and eukaryotes, METAnnotatorX2 provides profiles corrected for species’ genome size based on a size table inferred by genomes included in the preprocessed databases. The normalization of the read-based classification is also a new METAnnotatorX2 feature developed to obtain an accurate view of the actual abundance between the identified microorganisms disregarding their genome size. In fact, the latter proportionally impacts on the abundance of DNA fragments undergoing sequencing, thus introducing a bias in the proportion of reads obtained for each microbial species. In addition, this newly included approach has the advantage of being suited to analysis of shallow metagenomic data sets (or a subset of larger data sets), thus allowing high taxonomic accuracy with substantially reduced sequencing costs.

An option is provided to align reads against two databases for functional classification, i.e., the CAZy database (26, 27) and the MetaCyc database (28–30). Through these databases, METAnnotatorX2 generates an annotation profile of enzymes belonging to the glycobiome, classified in families as defined by the CAZy database (26, 27), and a profile of metabolic pathways based on similarities to enzymes present in the MetaCyc database (28–30).

Regarding assembly-based analyses, METAnnotatorX2 offers the possibility to perform metagenomic assemblies of short or long reads, as well as a hybrid mode option. The software (meta)SPAdes (14) is exploited to perform metagenomic assemblies of Illumina and/or Nanopore raw sequencing data (see Materials and Methods for details). Assembled contigs of >5 kbp are taxonomically classified through the preprocessed databases that are also used for read classification. In detail, megaBLAST is used to align assembled contigs to the preprocessed databases in order to taxonomically assign a given sequence in the same manner as performed for reads, and each pool of contigs sharing the same taxonomy is used to generate a GenBank file with genes predicted and functionally annotated. These GenBank files can be visually examined with genome browsers such as Artemis (31), thereby allowing straightforward genomic analysis of metagenomic contigs belonging to the same species. The implementation of this novel methodology for the classification of assembled contigs allows replacement of the previous gene-based system of METAnnotatorX, which relies on unprocessed RefSeq databases of genes. Thus, METAnnotatorX2 takes advantage of the full length of each assembled contig instead of shorter sequences represented by the genes encoded across contigs.

While METAnnotatorX2 has been developed to provide easy access to advanced metagenomic analyses to nonspecialized users, a wide range of settings can be edited in the “parameters” file, as described in the user manual, to obtain fine tuning of the analysis pipelines. All information needed to install and run METAnnotatorX2 is described in the user manual. METAnnotatorX2, its user manual, a test package encompassing Illumina and Nanopore data of three biological matrices, along with precomputed results and the set of preprocessed databases used by METAnnotatoX2 are available at http://probiogenomics.unipr.it/cmu/.

### The METAnnotatorX2 databases.

Decades of unsupervised submission of complete and draft microbial genomes to public repositories have led to iterative misclassifications that preclude accurate taxonomic profiling (20). For this reason, we released a set of databases of reference genomes whose taxonomy was checked and corrected to maximize the accuracy of homology-based taxonomic classification of reads and contigs. These databases are meant to be used in conjunction with METAnnotatorX2 and will be regularly updated on http://probiogenomics.unipr.it/cmu/. The previous release of the METAnnotatorX tool was used in conjunction with several gene-based databases directly downloaded from the NCBI server, including numerous entries afflicted by taxonomic classification errors. Therefore, in this new release of the tool, in addition to the novel strategy used for taxonomic classification of DNA sequences, we provide the user error-free databases that will improve the accuracy of taxonomic profiling.

Specifically, the prokaryotic database is generated by processing NCBI RefSeq genomes, whose taxonomic classification was corrected based on the ANI matrix precomputed by NCBI (20). For each species, each genome is classified as the closest reference genome if the ANI value is >94% with a subject and query coverage of >70%. This approach represents the gold standard for genome-based, species-level classification of genomes, as proposed by recent publications (25, 32, 33). The minimum query coverage is generally set at 70% because genomes undergoing classification through the use of reference genomes may represent draft sequences and/or encompass unique genomic regions, such as those originating from horizontal gene transfer (HGT) events, which are not considered in an ANI evaluation for genome-based species-level taxonomic classification (25, 32, 33). In this context, Richter and Rosselló-Móra (25) and others (32, 33) demonstrated that 20% of the genome is sufficient for accurate taxonomic classification through ANI evaluation. If the closest reference genome exhibits an ANI value of <94% yet shares the same genus as the original taxonomy attributed to the processed genome entry, its taxonomy is retained since a reference for that species is probably not defined (this regularly happens for newly discovered species). It is worth mentioning that in the current release of the METAnnotatorX2 databases (October 2020), out of 730,679 genomes checked by ANI, the number of genomes with a species match with the closest reference genome is 605,056. Thus, 125,623 genomes appear to have discrepancies in their assigned taxonomy and were reclassified based on the best ANI hit (>94%) using the NCBI reference genomes following the approach described in the guidelines provided by NCBI (20).

In order to reduce database redundancy, and consequently reduce computing times of METAnnotatorX2, we excluded from the prokaryotic database all microbial genomes with ANI of >99% with their respective species reference genome. Furthermore, the viral database has been generated using all genomes available in the NCBI RefSeq database with the inclusion of host information, while the fungal/protist eukaryotic database was designed to include all relevant genomes identified as “reference genomes” in the NCBI genome database. Notably, genomic contigs shorter than 1 kbp as well as 5 kbp and 12 kbp were removed from the viral, prokaryotic, and eukaryotic databases, respectively. Moreover, since we noticed that a number of microbial genome assemblies contained portions of “alien” DNA, we removed contigs with high sequence identity to common hosts of microbial populations, i.e., Homo sapiens, Bos taurus, Canis lupus, Equus caballus, Felis catus, *Gallus*, Mus musculus, Rattus norvegicus, and Sus scrofa. After these filtering steps, the resulting databases (release October 2020) are composed of 2,746 eukaryotic genomes, 27,463 prokaryotic genomes, and 10,419 viral genomes. In addition, ready-to-use databases for functional profiling based on CAZy (26, 27) and MetaCyc (28–30) classifications are available for download at http://probiogenomics.unipr.it/cmu/.

In order to include newly released microbial genomes, we intend to update all mentioned databases every 6 months.

### Performance assessment of taxonomic and functional profiling through read-based analysis of artificial data sets.

The performance of METAnnotatorX2 in read-based analyses was evaluated through artificial metagenomic data sets mimicking different biological matrices (see Data Set S1 in the supplemental material). These data sets were generated from the inclusion of viral, archaeal, bacterial, and eukaryotic (fungi) genomes typically found in a wide range of different environments such as cheese, human feces, soil, infant gut, lung, sputum, milk, skin, and vaginal swabs (Data Set S1). These genomes were processed using WGSIM software, included in the SAMtools suite (34), in order to obtain artificial 150-bp Illumina paired-end reads with a base error rate of 0.02 (https://github.com/lh3/wgsim/blob/master/wgsim.c#L248). A variable number of reads for each genome were shuffled in the same .fastq file in order to obtain artificial paired-end data sets with a known taxonomic composition (Data Set S1). For each matrix, we generated a data set consisting of eukaryotic (fungi), bacterial, archaeal, and viral genomes, representing a total of 20 million (20M), 10M, 1M, and 0.5M base pairs, respectively (Data Set S1).

10.1128/mSystems.00583-21.7 DATA SET S1 Taxonomic profiles expected and obtained by analysis of the nine artificial data sets using METAnnotatorX2, Kraken 2, MetaPhlAn 3, or METAnnotatorX along with a bar plot summary representation of all retrieved taxonomic profiles. Performance of METAnnotatorX2 in the prediction of the glycobiome and metabolic pathways of the nine artificial data sets. Results were obtained from assembly analysis of the nine artificial data sets, subdivided for taxonomic kingdom, including Illumina paired-end only, Nanopore-only, or hybrid assemblies, along with a summary table of the obtained results. Download Data Set S1, XLSX file, 5.1 MB. Copyright © 2021 Milani et al. 2021 Milani et al. https://creativecommons.org/licenses/by/4.0/ This content is distributed under the terms of the Creative Commons Attribution 4.0 International license.

**FIG 2:**
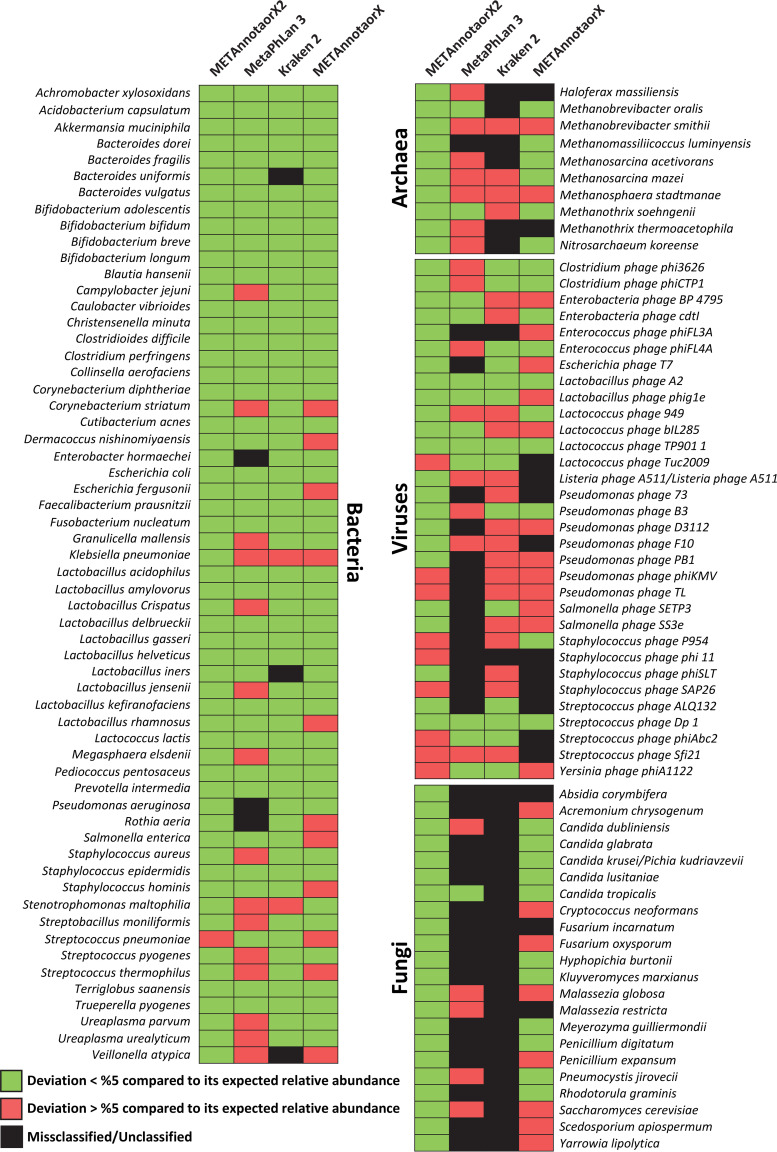
Evaluation of species-level classification accuracy based on analysis of the nine artificial data sets. The four different heatmaps show the performance of METannotatorX2, MetaPhlAn 3, Kraken 2, and METAnnotatorX tools in profiling the species used to generate the nine artificial data sets. The black color indicates that the species was undetected or misclassified, the green color means that the species was profiled with a deviation of <5% compared to its expected relative abundance. The red color means that the species was profiled with a deviation of >5% compared to its expected abundance.

**FIG 3:**
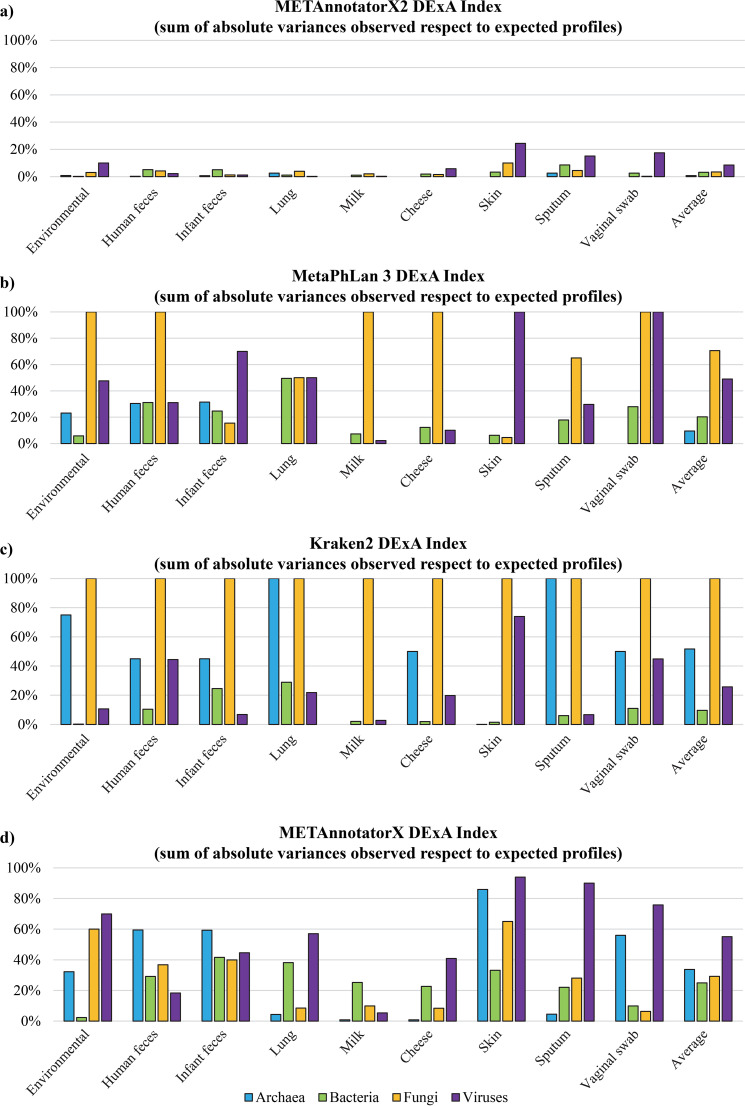
Evaluation of METannotatorX2, MetaPhlAn 3, Kraken 2, and METAnnotatorX software tool performance in retrieving the taxonomic profiles expected for the nine artificial data sets. The performance of the software tools was evaluated using the DExA index, represented by the ratio between the sum of absolute variances observed compared to the expected profiles. This means that a 0% DExA index score indicates that the tool had retrieved all species with the expected abundance, whereas an DExA index score of 100% means that the profiling software unclassified or misclassified all species in the artificial data set. Panels a, b, c, and d show the performances of METAnnotatorX2, MetaPhlAn 3, Kraken 2, and METAnnotatorX, respectively.

The nine data sets were subjected to read-based taxonomic analysis with METAnnotatorX2, allowing reconstruction of taxonomic profiles at the genus and species level through processing of 100,000 reads, the suggested default number of reads (editable by the user, see manual for details) needed to obtain a reliable taxonomic profile of species present at >0.5% relative abundance and compatible with shallow metagenomic applications (11) (Data Set S1). Notably, METAnnotatorX2 was able to detect each expected species present in these artificial data sets (Fig. 2) (Data Set S1). Moreover, in order to provide an overall evaluation of discrepancies in the retrieved profiles, we implemented a specific index, called Deviation from Expected Abundance index (DExA index), that represents the sum of absolute deviation in relative abundance of each observed species compared to expected profiles as listed in Data Set S1 (Fig. 3). Thus, a DExA index of 100% means completely different profiles, while a value of 0% indicates the obtained relative abundancies fully match the taxonomic composition of the artificial data sets listed in Data Set S1. This index allows the user to easily compare the performance of various taxonomic tools in reconstructing expected microbial profiles for artificial data sets (Fig. 3 and Data Set S1). Specifically, METAnnotatorX2 can detect species-level profiles of archaea, bacteria, and fungi with an average DExA of 0.76%, 3.21%, and 3.41%, respectively, along with viruses with an average DExA of 8.51% (Fig. 3 and Data Set S1).

Results obtained with METAnnotatorX2 were compared with those retrieved using available software for read-based taxonomic classification. In detail, we tested two of the most widely used software tools for taxonomic profiling of shotgun metagenomics data such as the recently updated MetaPhlAn 3.0 (16) and Kraken 2 (35), which rely on mapping of reads on a database of species-specific marker genes or a customized repository of whole genomes, respectively (Data Set S1). In addition, the previous version of the software METAnnotatorX was used to compare its efficiency with that of the novel pipeline. Notably, by using 100,000 reads, these software approaches generated outputs with mixed efficiency in reconstructing the expected taxonomic profiles, both in terms of misclassification and relative abundance accuracy (Fig. 2; see also Data Set S1), an issue that has previously been reported (19). As shown in Fig. 2, METAnnotatorX2 allowed the detection of all 123 microbial species present in the artificial data set, of which 91.9% had an observed relative abundance that deviated <5% from the expected relative abundance. In contrast, MetaPhlAn 3.0, Kraken 2, and METAnnotatorX were able to identify 72.4%, 73.2%, and 87.8%, respectively, of the species present in the artificial data set. Moreover, just 43.9%, 55.3%, and 61.0% of the species were profiled by MetaPhlAn 3.0, Kraken 2, and METAnnotatorX, respectively, with a relative abundance that showed a deviation of <5% from the expected profiles (Fig. 2; see also Data Set S1). Furthermore, the average DExA index values observed for species-level classification of viruses, archaea, bacteria, and fungi were higher than those obtained when employing METAnnotatorX2 (Fig. 3). In detail, the average DExA values observed for METAnnotatorX2, MetaPhlAn 3.0, Kraken 2, and METAnnotatorX were, respectively, 8.5%, 49%, 25.7%, and 55.1% for viruses, 0.8%, 9.4%, 51.7%, and 33.7% for archaea, 3.2%, 20.3%, 9.6%, and 24.9% for bacteria, and 3.4%, 70.6%, 100%, and 29.2% for fungi (Fig. 3). These values illustrate the marked improvement in taxonomic profiling accuracy offered by METAnnotatorX2 and associated preprocessed databases as based on a sample set of just 100,000 reads. Statistics regarding METAnnotatorX2 computing times tested using 5M reads from a paired-end Illumina cheese sample data set processed with a workstation equipped with AMD Ryzen Threadripper 3,970 × 32 physical cores (64 threads) and 128 Gb RAM (random access memory) are reported in Table S2.

10.1128/mSystems.00583-21.5 TABLE S2 Statistics regarding METAnnotatorX2 computing times tested using 5M read paired-end Illumina cheese sample data set processed with a workstation equipped with AMD Ryzen Threadripper 3,970 × 32 physical cores (64 threads) and 128 Gb RAM. Download Table S2, XLSX file, 0.01 MB. Copyright © 2021 Milani et al. 2021 Milani et al. https://creativecommons.org/licenses/by/4.0/ This content is distributed under the terms of the Creative Commons Attribution 4.0 International license.

In summary, these results underscore that, although other read mapping-based approaches allow the user to obtain acceptable results with fast computing times, they elicited subpar performances in taxonomic profile reconstruction compared to METAnnotatorX2. It may be expected that the shallow metagenomics approach represents the future successor to 16S rRNA gene microbial profiling as the gold standard for cost-effective taxonomic profiling of microbial communities with species-level accuracy. The artificial data sets corresponding to nine biological matrices were also processed for functional profile reconstruction through METAnnotatorX2 in order to reconstruct the glycobiome, i.e., the profile of enzymes involved in the metabolism of carbohydrates, and the profile of metabolic pathways encoded by the microbiome as classified by the CAZy and MetaCyc databases (Data Set S1). To validate these results, reads corresponding to each genome used for artificial data set generation were analyzed alone with METAnnotatorX2, and the resulting functional profiles were compared to preprocessed ones available through CAZy (http://www.cazy.org/Genomes.html) and MetaCyc databases (https://metacyc.org/), generating fully compatible results.

### Performance assessment of the metagenomic assembly and genomic analysis pipeline through artificial data sets.

Reconstruction of genomes from shotgun metagenomics data, i.e., metagenomic assembly, has the potential to provide genomic information of specific species constituting complex microbial populations (13, 14, 36–38). Nevertheless, while available assemblers have now been optimized to provide reliable results when coassembling multiple genomes in metagenomic settings, the subsequent processing of metagenomic contigs, including taxonomic assignment and gene analysis, very much relies on user expertise due to the absence of an optimized comprehensive bioinformatic processing platform.

For this reason, we included in METAnnotatorX2 a specific pipeline for the assembly of metagenomic data through employment of SPAdes software (13, 14), taxonomic assignment of assembled contigs and genomic analysis on a per-species basis. This pipeline was tested using the same Illumina paired-end artificial data sets employed for validation of read-based analysis (Data Set S1), as well as synthetic data sets represented by simulated Nanopore long reads of 8 kbp with an error rate of 0.3% (Data Set S1). Moreover, due to the higher error rate that characterizes long read sequencing, a hybrid approach is suggested in order to increase assembly coverage and sequence integrity through addition of short read data and thus increasing assembly base calling accuracy. For this reason, METAnnotatorX2 is equipped with a specific pipeline for hybrid assembly of short and long reads that was used for processing of the artificial short and long read data sets. In this context, to perform assembly of long reads, METAnnotatorX2 includes the additional assembler software Canu, which was absent in the previous version of METAnnotatorX.

Contigs of >5 kbp obtained from short read-only, long read-only or hybrid assembly of viral, archaeal, bacterial, or fungal data sets were subjected to taxonomic classification with METAnnotatorX2. This exercise showed that METAnnotatorX2 is able to assign the correct taxonomy with species-level accuracy to 98.5% of the contigs (intended as the sum of total sequence length classified relative to the total sequence length assembled) based on Illumina assembly, 99.2% of the contigs based on Nanopore assembly and 94.2% of the contigs based on hybrid assembly (Data Set S1). Moreover, each pool of contigs sharing the same taxonomy are processed and grouped by METAnnotatorX2 in a single GenBank file with genes predicted and functionally annotated based on the NCBI RefSeq genome, ready for downstream genomic analyses. As expected, the percentage of genomes reconstructed and the number of contigs retrieved for each species are proportional to genome size and the number of reads constituting the artificial data sets as reported in Data Set S1. In this regard, the hybrid assembly approach provided the best performance in terms of number of species assembled and in terms of total contig length (Data Set S1).

### Validation of METAnnotatorX2 through real biological samples.

A set of five biological matrices covering human, animal, food and environmental microbiota, i.e., human sputum, human vaginal swab, dog feces, parmesan cheese, and cow litters, were subjected to Illumina NextSeq 500 and Nanopore MinION sequencing in order to validate METAnnotatorX2 with “real-life” data sets (Data Set S2). Intriguingly, by using 100,000 reads, analysis of short reads revealed that MetaPhlAn 3, Kraken 2, and METAnnotatorX software are less effective compared to METAnnotatorX2 in reconstructing species-level taxonomic profiles from complex metagenomic data (Fig. S1 and Data Set S2). In fact, for each of the five tested matrices, METAnnotatorX2 was able to detect a higher number of microbial species with relative abundance of >0.5% compared to MetaPhlAn 3 and Kraken 2 (Fig. S1 and Data Set S2). This is explained by the fact that biological samples contain strains that are not present in public genomic databases, as previously highlighted (19). Thus, local alignments followed by assessment of sequence homology performed by METAnnotatorX2 were shown to provide increased sensitivity compared to read mapping since the latter requires higher read sequence identity compared to genomes or marker genes constituting the database used for taxonomic classification. In addition, taxonomic classification employing the previous version of the software METAnnotatorX, based on local alignment of the best hit against unprocessed gene-based databases, showed inconsistent hits with respect to the METAnnotatorX2 profiles. The absence in the previous pipeline of dedicated steps aimed at removing host DNA resulted in the identification of Homo sapiens-related contamination. Furthermore, the absence of error-free taxonomic databases in METAnnotatorX produced many false positive hits caused by misclassification in the microorganism taxonomy, e.g., Escherichia coli, Salmonella enterica, Streptococcus pneumoniae, and Halolamina sediminis.

10.1128/mSystems.00583-21.1 FIG S1 Evaluation of species-level taxonomic classification accuracy based on analysis of the five “real-life” samples. The heatmap shows all species retrieved with an abundance of  >1% in at least one sample through the use of METAnnotatorX2, Kraken 2, MetaPhlAn 3, or METAnnotatorX. The black color indicates that a species was not detected, while a green color indicates that a species was detected with the relative abundance reported in the cell. Each pipeline used a total of 100,000 reads to perform the taxonomic analysis. Download FIG S1, PDF file, 0.1 MB. Copyright © 2021 Milani et al. 2021 Milani et al. https://creativecommons.org/licenses/by/4.0/ This content is distributed under the terms of the Creative Commons Attribution 4.0 International license.

10.1128/mSystems.00583-21.8 DATA SET S2 Sequencing database and taxonomic profiles obtained from analysis of the five real biological samples using METAnnotatorX2, Kraken 2, MetaPhlAn 3, and METAnnotatorX along with a bar plot representation of the results. A comparison of raw and postgenome size normalization of METAnnotatorX2 results is also reported. Results were obtained from assembly of five real biological samples. The assembly was performed using only Illumina reads or only Nanopore reads or employing a hybrid strategy. The table summarizes the results obtained. Download Data Set S2, XLSX file, 0.1 MB. Copyright © 2021 Milani et al. 2021 Milani et al. https://creativecommons.org/licenses/by/4.0/ This content is distributed under the terms of the Creative Commons Attribution 4.0 International license.

Furthermore, it should be mentioned that METAnnotatorX2 performs normalization of taxonomic profiles by genome size through a table of average total length of genomes present in the databases used for taxonomic classification. A comparison of raw and normalized taxonomic profiles is reported in Data Set S2.

The short and long reads obtained for these data sets were also subjected to short read-only, long read-only, and hybrid assembly, followed by contig classification (Data Set S2). The results confirmed what already had been observed for the analysis of artificial data sets, highlighting the higher performance of hybrid assembly versus Illumina-only or Nanopore-only assemblies (Data Set S2).

### Impact of the number of reads on the accuracy of taxonomic profiling and computing times.

In order to evaluate the correlation between the number of reads employed for taxonomic profiling and the accuracy of the results as well as computing times, we performed analysis of 200, 400, 600, 800, 1,000 (1K), 5K, 10K, 50K, 100K, 200K, 500K, 1M, 2M, 10M, and 20M reads of three data sets corresponding to a real biological sample of cheese, an artificial data set Milk generated from publicly available genomes, and an additional artificial data set. This data set was generated using sequencing data obtained from bacterial strains of known species isolated and sequenced in the framework of this study with Illumina 250-bp technology, named UG (Unpublished Genomes data set) (Data Set S3). Notably, since the genomes used to construct the latter data set are not deposited in NCBI and consequently are not present in current METAnnotatorX2 databases, the UG data set represents a simulation of a real sample suitable for further validation of species-level profiling accuracy. Benchmark analysis was conducted exploiting the DExA index calculated by comparison of retrieved profiles with respect to: (i) expected profiles of the two artificial samples; (ii) the profile obtained at 10M reads, i.e., the highest sequencing depth, for the cheese real sample.

10.1128/mSystems.00583-21.9 DATA SET S3 Expected compositions of UG and Milk artificial data sets and METAnnotatorX2 taxonomic evaluation at different depth levels. Results were obtained from taxonomic profiling analysis of UG and Milk artificial data sets as well as of the Real cheese data set through the use of METAnnotatorX2, at different sequencing depth levels and evaluated by using the DExA index. Benchmark of time and memory usage. Average run times retrieved for METAnnotatorX2, MetaPhlAn 3, Kraken 2, and METAnnotatorX exploiting the UG and Milk artificial data sets as well as the real cheese data set at different sequencing depths. Setup impact on taxonomic profiling. Results obtained from taxonomic profiling analysis of UG and Milk artificial data sets and real cheese data set using METAnnotatorX2 with different variables and their impact on the predicted DExA index. Download Data Set S3, XLSX file, 0.07 MB. Copyright © 2021 Milani et al. 2021 Milani et al. https://creativecommons.org/licenses/by/4.0/ This content is distributed under the terms of the Creative Commons Attribution 4.0 International license.

The obtained results showed that MetannotatorX2 is able to predict reliable profiles already at 10K reads, as evidenced by a low average DExA index of 2.46% (Fig. S2 and Data Set S3). Moreover, the DExA curve tends to reach a plateau after 100K reads, corresponding to an average of 2.39% (Fig. S2 and Data Set S3). Remarkably, these findings indicate that profiling accuracy provided by MetannotatorX2 reaches its optimal value at 100K reads, thus making it unnecessary to set greater sequencing depth levels which would only affect the overall sequencing cost without any appreciable gain in taxonomic profiling performances (Fig. S2 and Data Set S3). Oddly, the METAnnotatorX2 approach should not be used for profiling of complete deep shotgun metagenomics data sets, as it is unfeasible to analyze the overall sequencing output of next-generation sequencers, such as the 400M reads produced by a NextSeq Run, within a reasonable time.

10.1128/mSystems.00583-21.2 FIG S2 Evaluation of METannotatorX2 performance in retrieving the taxonomic profiles at different sequencing depths. The performance of the tools were evaluated using the DExA index, represented by the ratio between the sum of absolute variances observed compared to the expected profiles. This means that a 0% DExA index score indicates that the tool had retrieved all species with the expected abundance, whereas an DExA index score of 100% means that the profiling software unclassified or misclassified all species in the artificial data set. Panel a shows the performances observed for METAnnotatorX2 at 14 different sequencing depth for Milk and UG synthetic data sets, reported as a bar plot representation. Panel b shows the performance observed for METAnnotatorX2 at 14 different sequencing depths for Milk and UG synthetic data set trough line graph representation. Panel c shows the performance observed for METAnnotatorX2 at 11 different sequencing depths for a real cheese metagenome, with DExA index calculated and based on composition retrieved at 20M reads. Download FIG S2, PDF file, 0.09 MB. Copyright © 2021 Milani et al. 2021 Milani et al. https://creativecommons.org/licenses/by/4.0/ This content is distributed under the terms of the Creative Commons Attribution 4.0 International license.

METAnnotatorX2 running times and memory requirements were also evaluated and compared to those of Kraken 2, MetaPhlAn 3, and METAnnotatorX (Data Set S3). As expected, the drawback of the higher accuracy of METAnnotatorX2 is the increased computing time that makes it unsuitable for taxonomic profiling of data sets constituted by millions of reads. Nevertheless, METAnnotatorX2 was designed to specifically analyze shallow metagenomics data sets of 100K reads or lower, which we demonstrated to be sufficient for accurate taxonomic profiling (Fig. S2), which was validated using previously published data (10–12). Furthermore, comparison against its predecessor METAnnotatorX highlighted that METAnnotatorX2 is faster when used for classification of reads, taking just a tenth of the computing time at 10K reads and less than half the time at 100K reads (Data Set S3).

In this context, it is worth mentioning that METAnnotatorX2 is also able to perform functional analyses of deep metagenomic data sets (Data Set S1) with low computing times, i.e., <5 min per sample for 20M read data sets (Table S2), while also allowing metagenomic assembly with an average of 29 min per sample, taxonomic classification of predicted contigs with an average of 4 min per sample and species-specific GenBank file generation in approximately 10 min (Table S2).

### Impact of the number of microbial species on the accuracy of taxonomic profiling.

Five artificial data sets composed of random bacterial species ranging from 10 to 1,000, each represented by 100K 150-bp-long random artificial Illumina reads, were used to test for phylogenetic biases in the detection of microbial taxa (Data Set S4). Remarkably, results of the taxonomic classification with METAnnotatorX2 showed that all bacterial species were successfully detected by METAnnotatorX2 in each sample, with average numbers of reads of 9,934, 1,983, 982, 196, and 98 for each taxon being very close to the theoretical values of 10,000, 2,000, 1,000, 200, and 100 reads (Data Set S4). Thus, increasing the number of putative taxa from 10 to 1,000 in a data set of 100K reads does not appear to affect the accuracy of taxonomic profiling.

10.1128/mSystems.00583-21.10 DATA SET S4 Performance of METAnnotatorX2 in the classification of 10, 50, 100, 500, and 1,000 random species of bacteria. Tracking of the number of false positive (FP), false negative (FN), true positive (TP), and true negative (TN) results in the microbial classification of five data sets composed by 10 random species of bacteria. Download Data Set S4, XLSX file, 0.1 MB. Copyright © 2021 Milani et al. 2021 Milani et al. https://creativecommons.org/licenses/by/4.0/ This content is distributed under the terms of the Creative Commons Attribution 4.0 International license.

Additionally, five artificial data sets composed of 10 random bacterial species, each represented by 100K 150-bp-long random artificial Illumina reads, were used for model performance measures to track the number of false positive (FP), false negative (FN), true positive (TP), and true negative (TN) results in the microbial classification (Data Set S4). The classification was performed by using a custom NCBI RefSeq genome database in which strains belonging to five random bacterial species of each data set were removed (Data Set S4). This benchmark allowed us to query the database with 50K reads of predicted TP and 50K reads of predicted TN, resulting in an average TP rate of 98.1%, and an average TN rate of 97.9%. The TN rate showed that most of the reads belonging to species removed in the custom database resulted in the classification of an unknown species, highlighting an average FN rate of 2.1% between data sets (Table S3).

10.1128/mSystems.00583-21.6 TABLE S3 Model performance measures data of five data sets composed of 10 random bacterial species. Download Table S3, XLSX file, 0.01 MB. Copyright © 2021 Milani et al. 2021 Milani et al. https://creativecommons.org/licenses/by/4.0/ This content is distributed under the terms of the Creative Commons Attribution 4.0 International license.

Altogether, the results achieved from the above *in silico* benchmarks confirmed the absence of significant biases in the taxonomic classification of reads and the high accuracy and validity of the proposed pipeline.

### Benchmarking of the main variables involved in taxonomic assignment.

The METAnnotatorX2 pipeline can be customized by editing of the “parameters” file, which allows the user to personalize a range of variables described in the user’s manual. The “parameters” file provided along with METAnnotatorX2 is preconfigured with optimal balanced settings for taxonomic profiling, which were derived from extensive testing. In detail, the cutoffs corresponding to minimum alignment E value and minimum alignment coverage as well as the minimum identity for genus-level and species-level classification of reads were evaluated exploiting shallow metagenomic data corresponding to the two artificial Illumina data sets Milk and UG (Fig. S3 and Data Set S3). The impact of the tested variables on the predicted taxonomic profiles was assayed by evaluation of the DExA index compared to the expected profiles of Milk and UG artificial data sets.

10.1128/mSystems.00583-21.3 FIG S3 Impact of user-defined variables on read-based taxonomic profiling. The four graphs show the impact on the DExA index of the main variables that can be manually set by the user in the parameters file. For the two artificial data sets UG and Milk, the DExA index was evaluated by comparison of retrieved taxonomic profiles versus the expected ones. The tested variables are: E value, query coverage, identity cutoff for genus assignment, and identity cutoff for species assignment. Download FIG S3, PDF file, 0.07 MB. Copyright © 2021 Milani et al. 2021 Milani et al. https://creativecommons.org/licenses/by/4.0/ This content is distributed under the terms of the Creative Commons Attribution 4.0 International license.

Regarding the minimum E value for alignment hits, data collected revealed that the average DExA index is stable for values ranging from e−1 to e−40, indicating that the alignments’ best hit generally have higher E values (Fig. S3 and Data Set S3). In contrast, E values lower than e−40 induce an increase in the average DExA index due to excessive stringency that causes exclusion of false negative results (Fig. S3 and Data Set S3). Based on these results, the default minimum E value was set to e−5.

Furthermore, analysis of the minimum alignment coverage indicated limited impact of this setting on quality of the results, with 100% coverage providing limited accuracy gain in terms of average DExA index (Fig. S3 and Data Set S3). Ninety-five percent of coverage was chosen as the default setting in order to compensate for expected inaccuracy in the alignment of individual read ends.

Moreover, we also evaluated the impact of the minimum identity for taxonomic assignment at the genus and species level (Fig. S3 and Data Set S3). These analyses showed that the minimum identity for genus-level classification has limited impact on the predicted profiles. Nevertheless, since real biological samples may include novel species without representative genomes in public databases, we suggest the use of a minimum identity of 20% to allow for their classification at the genus level. In contrast, the minimum identity for species-level classification negatively affects the average DExA index for identity values higher than 94% due to excessive stringency, thus confirming previously reported data regarding genomic average nucleotide identity between strains of the same species (20, 32, 39). Thus, 94% identity is provided as the default setting as minimum identity for species-level classification.

### Conclusions.

Despite the increasing scientific interest in performing shallow metagenomics, functional profiling of metagenomes and hybrid assembly approaches, a comprehensive and ready-to-use bioinformatic platform for guided data analysis is still missing.

For these reasons, we established METAnnotatorX2, a user-friendly and highly customizable application designed to include all relevant tools for analysis of shotgun metagenomic data. These tools encompass read-based analyses for taxonomic profile reconstruction and functional assessment of metagenomes. Furthermore, short and long reads can be used as input for unmixed or hybrid assemblies, and the retrieved metagenomic contigs can be taxonomically classified and processed to obtain species-specific GenBank files with predicted and functionally annotated genes, ready for downstream genomic analyses. In the context of this study, we developed a set of preprocessed databases specific for viruses, prokaryotes, as well as fungi and protists that were designed to allow accurate taxonomic assignments of reads and contigs corresponding to complex microbial communities. In order to provide default settings for the most common usage scenarios, we performed testing of multiple settings of the main variables that can be defined by the user in order to customize METAnnotatorX2 pipeline.

Performances of METAnnotatorX2 were assessed and compared to two other available and commonly used software tools, as well as to the previous version METAnnotatorX, using artificial short and long read data sets mimicking microbial populations typically harbored by nine different matrices as well as by analysis of five “real-life” biological samples. Our results indicate that METAnnotatorX2 represents the preferred choice for taxonomic analysis of shallow metagenomics data sets as well as for functional analysis of microbiomes. In addition, METAnnotatorX2 provides a complete pipeline for unmixed and hybrid assemblies of short and long reads, providing species-specific reconstruction and gene annotation of microbial genomes from metagenomic data.

## MATERIALS AND METHODS

### Ethical approval and consent to participate.

All experimental procedures and protocols involving animals were approved by the Veterinarian Animal Care and Use Committee of Parma University and conducted in accordance with the European Community Council Directives dated 22 September 2010 (2010/63/UE). Human participants gave their informed written consent before enrollment. All investigations were carried out following the principles of the Declaration of Helsinki.

### Sample collection.

For the purpose of this study, a total of five samples were collected, encompassing five biological samples of each of the following matrices: animal feces, human vaginal swabs, human oral samples, litters from dairy cattle, and a cheese sample. Animal feces were collected immediately after defecation. Humans and animals included in this study had not taken antibiotics during the previous 6 months. The cheese sample was retrieved by trimming a fresh cheese shape. In all cases, immediately after collection, samples were kept on ice and shipped to the laboratory under frozen conditions where they were preserved at −80°C until further processing.

Human and animal fecal samples together with oral and litter samples were subjected to DNA extraction using the QIAmp DNA stool minikit (Qiagen, Germany). A ZymoBIOMICS DNA Miniprep kit (Zymo Research Corporation USA) was used for DNA extraction from vaginal swabs, while DNA extraction from cheese samples was performed using the DNeasy mastitis minikit (Qiagen, Germany). DNeasy PowerSoil kit (Qiagen, Germany) was employed for DNA extraction from soil samples. In all cases, DNA extractions were performed following the manufacturers’ instructions.

### Illumina shotgun metagenomics sequencing.

The extracted DNA was prepared following the Illumina Nextera XT DNA Library Preparation kit. Briefly, the DNA samples were enzymatically fragmented, barcoded, and purified involving magnetic beads. Samples were then quantified using a fluorometric Qubit quantification system (Life Technologies, USA), loaded on a 2200 Tape Station instrument (Agilent Technologies, USA) and normalized to 4 nM. Sequencing was performed using an Illumina NextSeq 500 sequencer with NextSeq High Output v2 kit Chemicals 150 cycles for metagenomics data sets and using an Illumina MiSeq sequencer with MiSeq reagent kit V3 600 cycles for bacterial genomes.

### Nanopore shotgun metagenomics sequencing.

DNA was extracted from matrices using different commercially available kits; the DNA from a vaginal swab sample was extracted with ZymoBIOMICS miniprep kit (Zymo Research, USA), from an oral sample with QIAmp DNA minikit (Qiagen, Germany), from a dog fecal sample and soil sample with the QIAamp Fast DNA Stool minikit (Qiagen, Germany). A quantity and quality check was performed using a fluorometric Qubit system (Life Technologies) and Agilent Tape Station. DNA was then processed following the Native barcoding genomic DNA (with EXP-NBD104, EXP-NBD114, and SQK-LSK109) protocol which consists of a few steps: DNA repair and end prep, native barcode ligation, adapter ligation and cleanup. At the end of the protocol, the fragment length and the amount of the pooled samples were verified by means of Agilent Tape Station and Qubit fluorometer. The sequencing was performed on a MinION (Oxford Nanopore Technologies) loading the final library on a 9.4.1 flow cell following the section “Priming and Loading the SpotON flow cell” of the previously mentioned protocol.

### Analysis of metagenomic data sets.

The obtained fastq files were filtered to remove reads with a quality of <25, and to retain reads with a length of  >149 bp. Quality-filtered data were then used for further analysis employing METAnnotatorX2, Kraken 2, MetaPhlAn 3, or METAnnotaorX for taxonomic profile reconstruction. Software packages used for comparisons were used with default settings. Further assembly analyses were performed with METAnnotatorX2 by means of the metagenomic assembler (meta)SPAdes with the running mode “spades.py –meta”.

### Availability of data and materials.

Artificial data sets used in this study can be downloaded at http://probiogenomics.unipr.it/cmu/. Real biological samples used in this study can be downloaded by NCBI trough BioProject code or accession no. PRJNA672826. Source code is available at https://doi.org/10.5281/zenodo.4954284.
