# Supplementary material for: METAnnotatorX2: a Comprehensive Tool for Deep and Shallow Metagenomic Data Set Analyses
Source: mSystems. 2021 Jun 29;6(3):e00583-21. doi: 10.1128/mSystems.00583-21 (PMC8269244; doi:10.1128/mSystems.00583-21)
Supplement: FIG S1 [file msystems.00583-21-sf001.pdf]

ID Species	METAnnotatorX2					METAnnotatorX					Kraken 2					MetaPhlan 3				
	Dog Faeces	Cheese	Cow Litter	Sputum	Vaginal Swab	Dog Faeces	Cheese	Cow Litter	Sputum	Vaginal Swab	Dog Faeces	Cheese	Cow Litter	Sputum	Vaginal Swab	Dog Faeces	Cheese	Cow Litter	Sputum	Vaginal Swab
Number of Retrieved species	10	6	27	24	12	13	2	11	21	17	23	1	8	4	2	3	6	6	4	3
Lactococcus lactis		89.04%					74.36%					84.70%					73.61%			
Homo sapiens									1.46%	2.36%	5.29%		1.38%	83.68%	93.63%					
Prevotella copri	36.88%					24.26%										92.86%				
Staphylococcus xylosus			5.56%					10.12%					21.99%					31.26%		
Lactobacillus iners					10.20%					5.66%										43.30%
Neisseria flavescens				0.69%					0.63%										57.01%	
Geoglossum unknown species				22.57%	28.52%															
Staphylococcus arlettae			11.09%					2.24%										35.53%		
Human endogenous retrovirus K																				40.23%
Saccharopolyspora rectivirgula			9.78%					6.80%										18.96%		
Lactobacillus jensenii										10.43%					4.85%					16.47%
Scortechinia unknown species				12.82%	16.08%															
Bacteroides unknown species	16.48%			0.76%		4.27%														
Porphyromonas somerae									1.87%										16.62%	
Streptococcus mitis				2.64%					1.17%					0.88%					12.27%	
Lactococcus virus bIL67																	15.15%			
Prevotella unknown species	9.19%			4.54%					1.29%											
Corynebacterium stationis			3.11%					2.35%					4.66%					4.54%		
Lactobacillus mulieris					14.25%															
Haemophilus sp HMSC71H05																			14.11%	
Virus Poxviridae unknown species				5.43%	6.23%				0.98%	1.38%										
Phocaeicola unknown species	13.00%																			
Echinosphaeria unknown species				5.15%	6.23%															
Corynebacterium unknown species			9.79%					1.50%												
Salmonella enterica									4.30%	6.80%										
Lactobacillus diolivorans		4.85%					3.54%										2.20%			
Faecalibacterium prausnitzii						3.10%					7.40%									
Staphylococcus pseudoxylosus			10.05%																	
Bacteroides plebeius						4.56%										5.22%				
Staphylococcus unknown species			5.15%					4.59%												
Weissella hellenica			2.70%					2.03%										4.88%		
Hemileia unknown species			9.29%																	
Mycobacterium unknown species									3.13%	4.66%										
Bacteroides vulgatus						1.18%					6.57%									
Fusobacterium unknown species	7.56%																			
Escherichia coli									2.95%	4.40%										
Haemophilus parainfluenzae				3.91%					1.47%					1.90%						
Streptococcus pneumoniae									3.02%	4.14%										
Bacteroides coprocola						4.78%										1.92%				
Prevotella melaninogenica				2.27%					1.35%		1.54%			1.43%						
Bertia unknown species				2.91%	3.39%															
Pasteurella unknown species				2.57%	3.52%															
Paenibacillus odorifer									2.32%	3.53%										
Plasmodium unknown species				2.51%	3.09%															
Chaetosphaerella unknown species				2.40%	2.90%															
Neisseria subflava				3.27%										1.83%						
Porphyromonas unknown species				3.70%					1.39%											
Bacteroides salanitronis						0.92%					4.15%									
Sulfolobus acidocaldarius									1.98%	2.90%										
Salmonella virus FelixO1																		4.84%		
Tympanopsis unknown species				2.28%	2.56%															
Phocaeicola coprocola	4.57%																			
Bacteroides fragilis						1.15%					3.29%									
Halolamina sediminis									3.49%	0.84%										
Phocaeicola plebeius	3.90%																			
Streptomyces unknown species			3.89%																	
Enterobacter hormaechei									1.44%	2.39%										
Corynebacterium casei			0.92%					0.97%					1.89%							
Prevotellaceae unknown species									3.75%											
Prevotella nanceiensis				2.43%					1.25%											
Fusobacterium perfoetens						3.57%														
Faecalibacterium unknown species	3.54%																			
Shigella sonnei									1.43%	2.04%										
Bacillus cereus									1.36%	2.10%										
Lactobacillus phage Lrm1																	3.40%			
Fusobacterium mortiferum						0.65%					2.56%									
Corynebacterium xerosis			0.90%					0.68%					1.57%							
Bacteroides thetaiotaomicron						0.63%					2.46%									
Paraprevotella xylaniphila						0.77%					2.30%									
Pan troglodytes									1.14%	1.83%										
Sutterella unknown species	1.31%					1.65%														
Acinetobacter baumannii									1.17%	1.74%										
Brachybacterium unknown species			2.33%					0.53%												
Lactobacillus phage JCL1032																	2.78%			
Bacteroides caecimuris						0.51%					2.14%									
Bacteroides mediterraneensis						2.59%														
Ruania unknown species			2.57%																	
Dietzia timorensis								1.17%					1.36%							
Lactobacillus phage J1																	2.46%			
Bacteroides coprophilus						2.40%														
Saccharopolyspora unknown species			2.35%																	
Lactobacillus paracasei		1.14%					0.64%					0.57%								
Brevibacterium linens								0.53%					1.74%							
Lactobacillus unknown species		1.30%								0.95%										
Prevotella ruminicola											2.22%									
Ruania albidiflava								2.16%												
Lactobacillus kefiri		1.30%					0.85%													
Geoglossum glabrum				0.96%	1.11%															
Neisseria unknown species				1.09%					0.95%											
Sphingomonas unknown species			2.04%																	
Haemophilus unknown species				1.34%					0.68%											
Nomascus leucogenys									0.77%	1.23%										
Corynebacterium glyciniphilum								1.32%					0.66%							
Fusobacterium ulcerans						0.53%					1.44%									
Butyricimonas faecalis											1.96%									
Bacteroides ovatus						0.77%					1.17%									
Haemophilus haemolyticus				1.93%																
Dietzia unknown species			1.79%																	
Chlorocebus sabaeus									0.76%	1.03%										
Prevotella intermedia											1.75%									
Prevotella aurantiaca				1.14%					0.54%											
Brevibacterium unknown species			1.67%																	
Lactococcus unknown species		1.63%																		
Bacteroides cellulosilyticus											1.58%									
Bacteroides caccae											1.57%									
Bacteroides barnesiae						1.53%														
Bacteroides helcogenes											1.50%									
Prevotella oris											1.49%									
Paracoccus alcaliphilus			1.48%																	
Bacteroides dorei											1.47%									
Microbacterium unknown species			1.45%																	
Aspergillus unknown species			1.45%																	
Janibacter unknown species			1.37%																	
Parabacteroides unknown species	1.37%																			
Fusobacterium varium											1.33%									
Neisseria cinerea				1.32%																
Aegilops tauschii								1.32%												
Streptococcus unknown species				1.29%																
Massilia unknown species			1.28%																	
Hemileia vastatrix			1.27%																	
Sphingobacterium unknown species			1.26%																	
Paracoccus unknown species			1.24%																	
Prevotella fusca											1.24%									
Veillonella rogosae				1.19%																
Pedobacter unknown species			1.17%																	
Bacteroides massiliensis						1.17%														
Morococcus cerebrosus				1.16%																
Aerococcus urinaeequi													1.11%							
Nocardioides unknown species			1.07%																	
Weissella unknown species			1.02%																	
Sutterella megalosphaeroides											1.00%									

Figure S1
