# Supplementary material for: METAnnotatorX2: a Comprehensive Tool for Deep and Shallow Metagenomic Data Set Analyses
Source: mSystems. 2021 Jun 29;6(3):e00583-21. doi: 10.1128/mSystems.00583-21 (PMC8269244; doi:10.1128/mSystems.00583-21)
Supplement: FIG S2 [file msystems.00583-21-sf002.pdf]

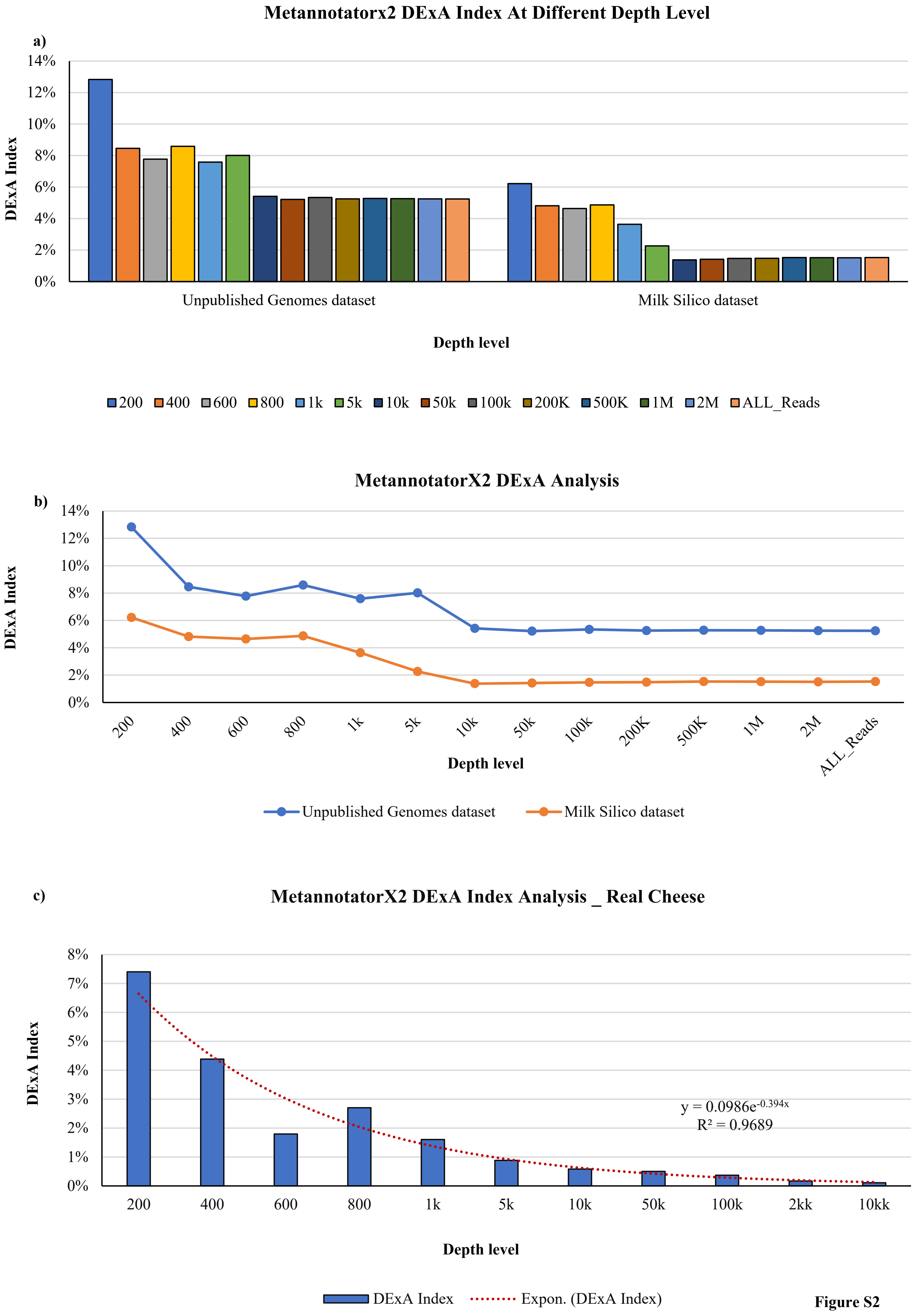
Metannotatorx2 DExA Index At Different Depth Level
a)
DExA Index
14%
12%
10%
8%
6%
4%
2%
0%
Unpublished Genomes dataset
Milk Silico dataset
Depth level
200 400 600 800 1k 5k 10k 50k 100k 200K 500K 1M 2M ALL_Reads
MetannotatorX2 DExA Analysis
b)
DExA Index
14%
12%
10%
8%
6%
4%
2%
0%
200 400 600 800 1k 5k 10k 50k 100k 200K 500K 1M 2M ALL_Reads
Depth level
Unpublished Genomes dataset
Milk Silico dataset
c)
MetannotatorX2 DExA Index Analysis _ Real Cheese
DExA Index
8%
7%
6%
5%
4%
3%
2%
1%
0%
$y = 0.0986e^{-0.394x}$
$R^2 = 0.9689$
200 400 600 800 1k 5k 10k 50k 100k 2kk 10kk
Depth level
DExA Index
Expon. (DExA Index)

Figure S2
