# Supplementary figures and images for: METAnnotatorX2: a Comprehensive Tool for Deep and Shallow Metagenomic Data Set Analyses
Source: mSystems. 2021 Jun 29;6(3):e00583-21. doi: 10.1128/mSystems.00583-21 (PMC8269244; doi:10.1128/mSystems.00583-21)

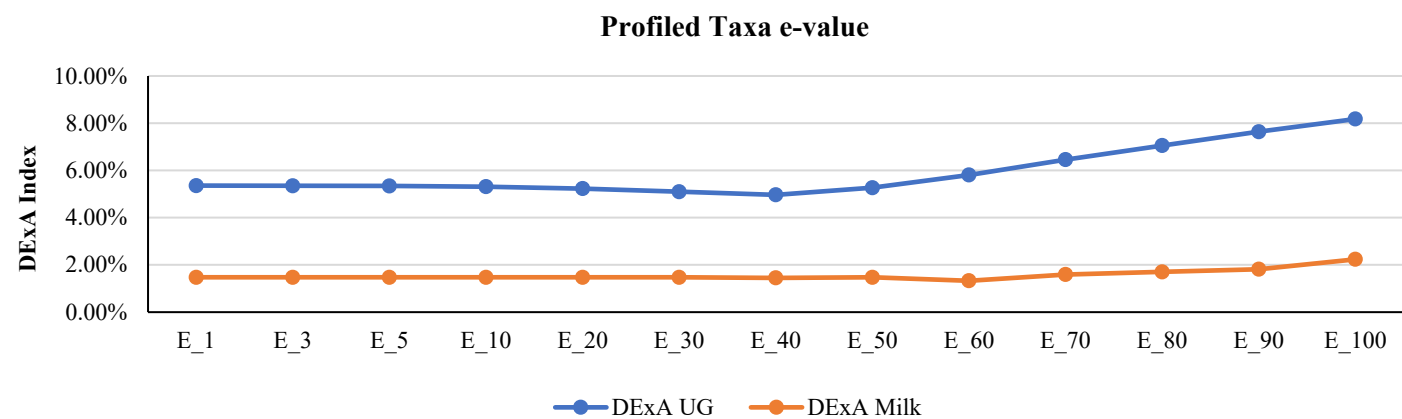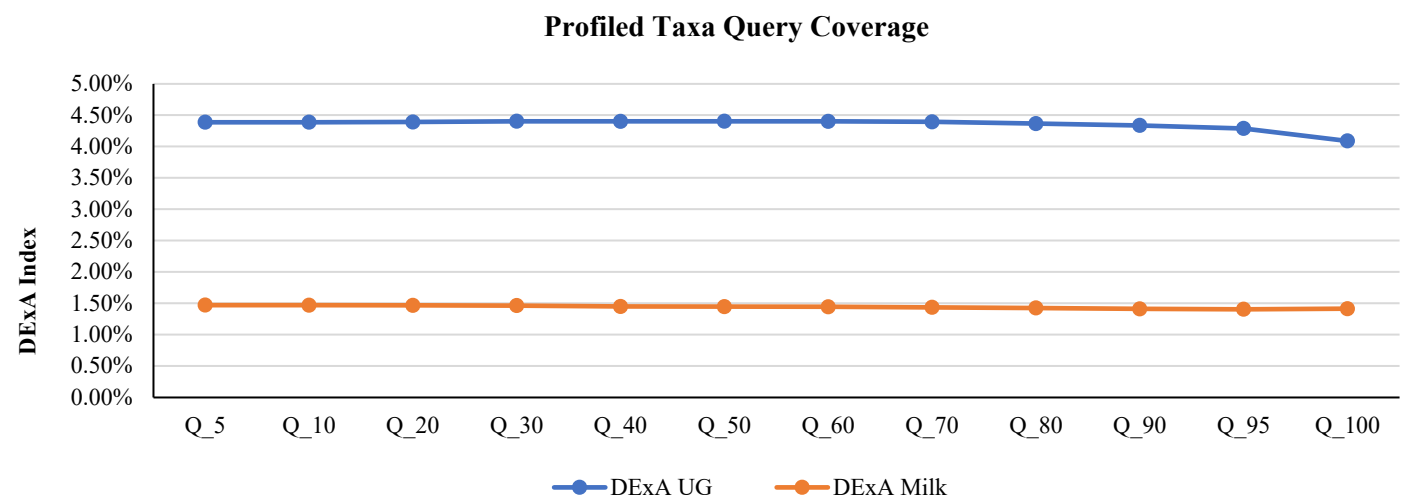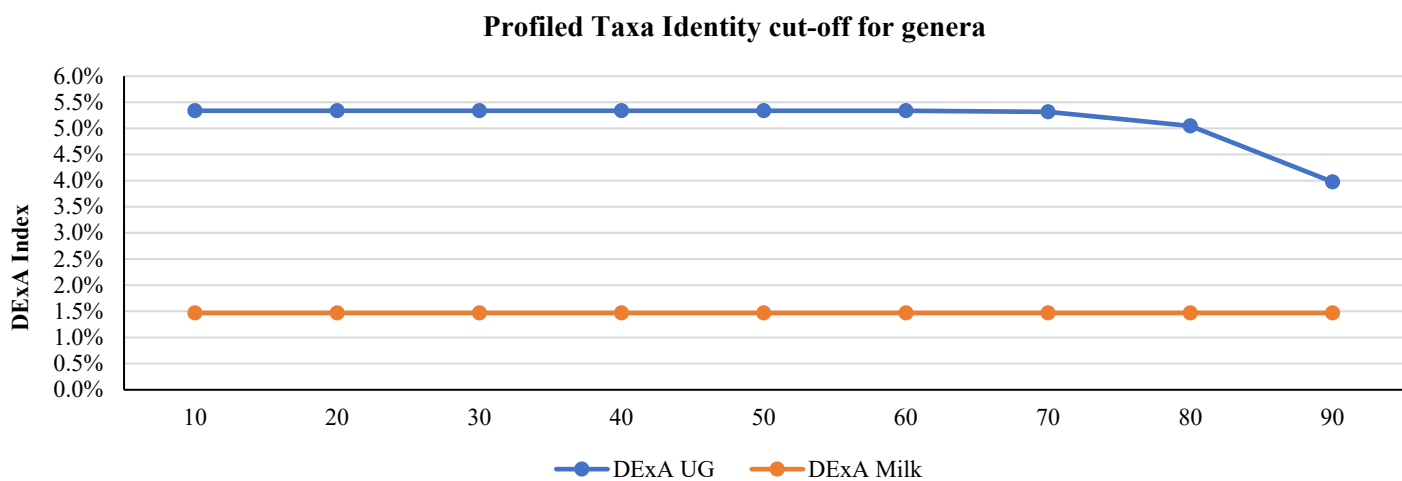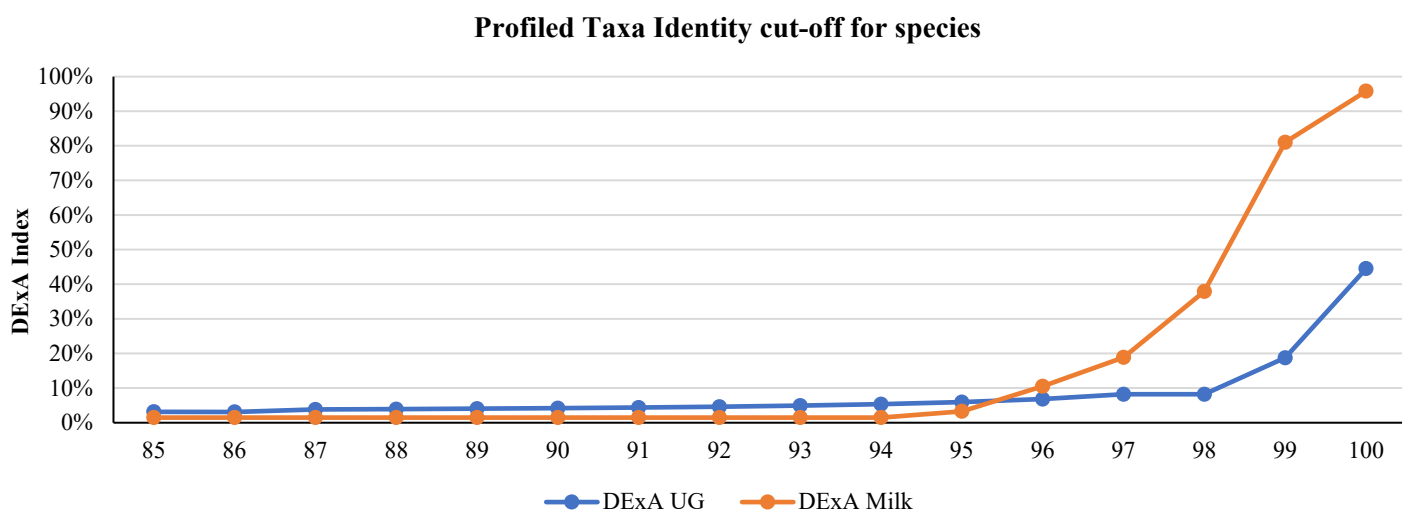

Figure S3

Supplement: FIG S3 [file msystems.00583-21-sf003.pdf]
